# Effect of Graphene/Spherical Graphite Ratio on the Properties of PLA/TPU Composites

**DOI:** 10.3390/polym14132538

**Published:** 2022-06-22

**Authors:** Zenghui Yang, Haihua Wu, Renjing Zhang, Kaixin Deng, Yan Li, Zhi Liu, Qiang Zhong, Yi Kang

**Affiliations:** Hubei Engineering Research Center for Graphite Additive Manufacturing Technology and Equipment, China Three Gorges University, Yichang 443002, China

**Keywords:** PLA/TPU composites, graphene/spherical graphite ratio, FDM, microwave absorption performance, mechanical property

## Abstract

Wave-absorbing materials are developing in the direction of “light weight, wide frequency band, thin layer and high strength”, and it is difficult to achieve the synergy between wave-absorbing performance and mechanical properties when graphene absorbent is compounded with a single resin matrix. In this paper, based on the preparation of a new composite absorbing wire with a graphene (GR)/spherical graphite (SG) double absorbent and polylactic acid (PLA)/thermoplastic polyurethane (TPU) double matrix, we proposed a new method to prepare samples for testing the electromagnetic parameters and tensile strength by fused deposition modeling (FDM). Furthermore, the effect of SG/GR ratio on the microwave absorbing properties and mechanical properties of PLA/TPU composites was specifically studied. It was found that when the ratio of SG/GR was small (0:5, 1:4), the dielectric loss (interfacial polarization loss, dipole polarization loss, conductivity loss) and attenuation ability of the composites were stronger, and the impedance matching was better. When the SG/GR ratio was large (5:0, 4:1), the composites had high strength and toughness. When the ratio of SG/GR was moderate (2:3, 3:2), it could retain the absorbing and mechanical properties of the absorbing materials. On the one hand, the SG and PLA/TPU matrix formed an “island structure”, which improves the dispersion of GR; on the other hand, the GR and PLA/TPU matrix formed a “core-shell structure”, which promotes polarization and multiple scattering.

## 1. Introduction

With the advent of the 5G era, electronic communication and radar detection technology are widely used in military and civil fields, which bring convenience to human life. At the same time, the resulting electromagnetic radiation problem is becoming more and more serious, and the development of wave-absorbing materials is imminent [1,2,3]. At present, carbon materials have extraordinary application potential for sensors, biology, energy, environment and other fields [4,5,6]. Because of their light weight, strong conductivity, good stability and other advantages, they can effectively replace traditional wave absorbing materials [7]. However, carbon-based materials (such as spherical graphite (SG), carbon black (CB), carbon fiber (CF), carbon nanotubes (CNTs), graphene (GR), etc.) mostly exist in the form of powder and fiber, which are difficult to form, and the impedance matching level is low when used alone. Therefore, researchers often combine them with various matrices (such as epoxy resin (EP), polypropylene (PP), polyoxyethylene (POE), polylactide (PLA), thermoplastic polyurethane (TPU), etc.) to improve the molding process, and adjust the impedance matching by reducing the electrical conductivity, which makes it possible to design and manufacture complex and efficient wave absorbers based on carbon materials.

Graphene (GR), as a carbon allotrope, is generally prepared by mechanical stripping, oxidation-reduction and chemical deposition [5]. It has excellent conductivity, usually used as an electrode material because of its two-dimensional porous structure and large specific surface area, and is expected to become the most ideal high-efficiency absorbent [8,9]. Xue [10] mixed a graphene oxide aqueous dispersion with liquid epoxy resin by wet transfer-three-roll grinding and prepared a graphene oxide/epoxy resin composite by the mold casting process, which obtained good dispersion and wave absorption effect. However, the temperature was difficult to control and a special mold needed to be developed which led to high costs. Liu [11] developed GR/PLA composite wires with different contents, and then realized the rapid preparation of porous absorbers by FDM technology. The effective absorption bandwidth reached 6.7 GHz and the maximum reflection loss was −33 dB. However, when the GR content was high, its strength and toughness were insufficient.

In recent years, researchers have mostly focused on the broadband and strong absorption of absorbing materials, but paid little attention to the mechanical properties of practical engineering applications. On the choice of matrix, He [12] and others prepared PLA/TPU nanofiber composites with in situ ordered orientation by the melt blending method. The elongation at break was about 12 times that of pure PLA and reached 113% [13], which greatly improved the brittleness of the single PLA matrix. On the choice of filler, Yu [14] filled graphene nanosheets/nano-SiO_2_ hybrid particles into an epoxy resin matrix. He found that, compared with a single filler, binary hybrid particles had the best reinforcing and toughening effect on composites.

In this paper, GR and SG were selected as binary carbon-based absorbents. After preparing SG/GR/PLA/TPU composite wires with six SG/GR ratios (5:0, 4:1, 3:2, 2:3, 1:4, 0:5) by the two-step method [3], coaxial rings and tensile samples were quickly prepared by FDM technology. Firstly, the microstructure, lattice structure, chemical bonds and functional groups of SG and GR were characterized by SEM, TEM and FTIR. The phase structure and graphitization degree of the composites were further analyzed by XRD and Raman spectroscopy. Secondly, the mechanism of dielectric loss was revealed by electromagnetic parameter test. The reflectivity, attenuation constant and value were calculated by the formula, and the influence of SG/GR ratio on the microwave absorbing properties of the composites was studied. Finally, based on the tensile test and the micro-morphology of the composite powder, the influence of SG/GR ratio on the mechanical properties of the composite was studied by analyzing the dispersion state of SG and GR in PLA/TPU, which provided a reference for high strength and high toughness absorbing materials.

## 2. Experimental Section

### 2.1. Materials and Instruments

The raw materials and instruments required for the experiment are shown in Table 1 and Table 2, respectively.

### 2.2. Preparation of SG/GR/PLA/TPU Composites

**Figure 1:**
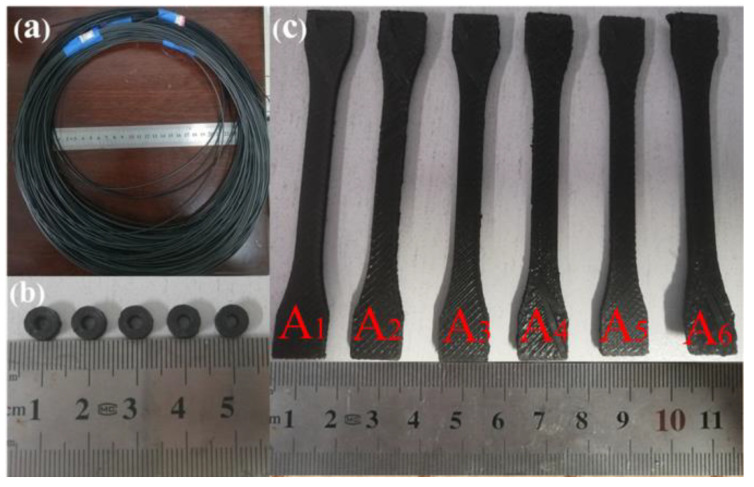
(**a**) SG/GR/PLA/TPU composite wire; (**b**) electromagnetic parameter sample; (**c**) tensile specimens of A_1_~A_6_ composites.

PLA and TPU powders were dried in an electrothermal constant-temperature blast drying oven at 60 °C for 6 h before being used, then mixed with SG and GR powders and zirconia beads in a horizontal planetary ball mill at a mass ratio of 1:1 for 3.5 h to obtain composite powders with different contents. Then, a single-screw extruder (130 °C in the first zone of the barrel, 150 °C in the second zone of the barrel, 170 °C in the die mouth, 16 r/min in the screw speed and 8 r/min in the traction speed) was used to prepare the corresponding 3D printing composite wire (Figure 1a, the wire diameter is Φ1.75 ± 0.05 mm), and its formula composition is shown in Table 3.

The composite wire rods were printed with double nozzle printer (nozzle temperature was 210 °C, platform temperature 50 °C, printing speed 30 mm/s, filling rate 100%) to prepare electromagnetic parameter samples and tensile samples. The electromagnetic parameter sample was a coaxial ring with an outer diameter of 7 mm, an inner diameter of 3.04 mm and a thickness of 2.5 mm, as shown in Figure 1b. Tensile specimen was the 1BA specimen of GB/T 1040.1-2006 standard, as shown in Figure 1c.

### 2.3. Performance Characterization

The methods for testing and characterizing the properties of SG, GR and SG/GR/PLA/TPU composites are shown in Table 4 below

## 3. Results and Discussion

### 3.1. Material Characterization Analysis

#### 3.1.1. The Micro-Morphology and Size Structure of SG and GR

**Figure 2:**
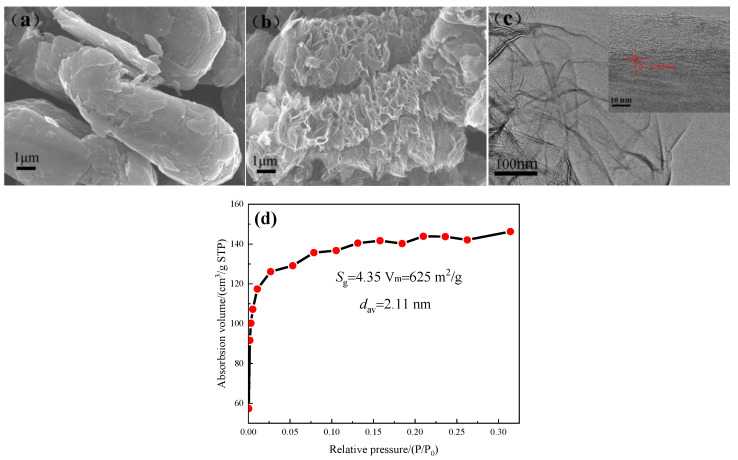
(**a**) SEM of SG powder; (**b**) SEM of GR powder; (**c**) TEM of GR powder; (**d**) N_2_ adsorption-desorption isotherm of GR powder.

**Figure 3:**
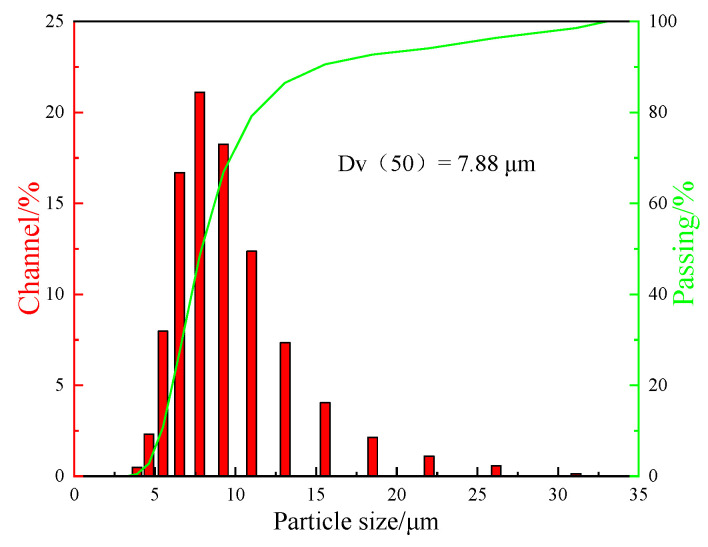
Particle size distribution diagram of SG powder.

The micro-morphology of SG and GR powders were as shown in Figure 2a,b, showing three-dimensional elliptical spheres (smooth surface) and a two-dimensional curled sheet (rich surface folds), respectively. GR was stacked by 10 graphene layers with a sheet diameter of about 5 μm (lattice structure is shown in Figure 2c). The measured specific surface area was 625 m^2^/g and the average pore diameter was 2.11 nm (adsorption-desorption isotherm is shown in Figure 2d). The particle size distribution of SG measured by laser particle size analyzer is shown in Figure 3, and its average particle size was 7.88 μm.

**Figure 4:**
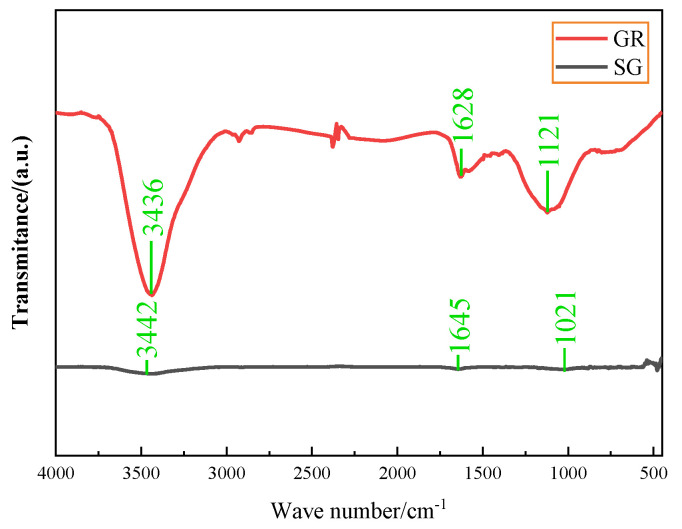
Fourier infrared spectra of SG and GR powders.

Figure 4 shows the infrared spectra of SG and GR powders in the wave number range of 4000~450 cm^−1^. There were certain absorption peaks at 3442 cm^−1^ and 3436 cm^−1^, 1645 cm^−1^ and 1628 cm^−1^, 1021 cm^−1^ and 1121 cm^−1^, which corresponded to the stretching vibration peaks of -OH, C=C, -C-O-C, respectively [15]. Among them, the peak intensity of GR was very strong and SG was very weak, which indicated that there were still a lot of oxygen-containing functional groups such as -OH, -C-O-C in the GR after redox.

#### 3.1.2. Phase Structure and Graphitization Degree of SG/GR/PLA/TPU Composites

**Figure 5:**
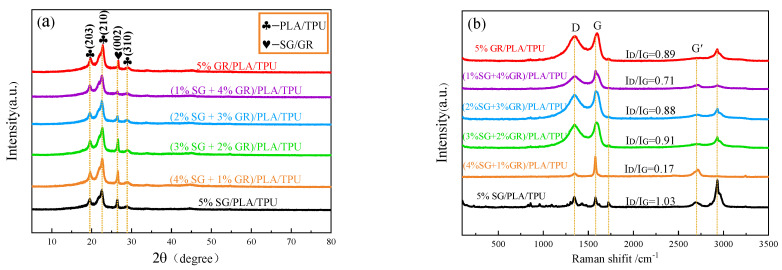
Six SG/GR/PLA/TPU composites with different contents: (**a**) XRD; (**b**) Raman spectrum.

Figure 5a shows the XRD patterns of six SG/GR/PLA/TPU composites with different contents. Diffraction peaks belonging to PLA/TPU were observed at 2θ = 19.55°, 22.62° and 28.90°, corresponding to (203), (210) and (310) crystal planes, respectively. The C-phase (SG/GR) diffraction peak corresponding to (002) crystal plane (JCPDSNo. 41-1487) was observed at 2θ = 26.46°. The difference is that the SG/GR peak of (1% SG + 4% GR)/PLA/TPU composite is the smallest, which promotes the crystallization of PLA/TPU. The SG/GR peak of (4% SG + 1% GR)/PLA/TPU composite is closer to the peak intensity of the (210) crystal plane, which is beneficial to the uniform composite of a PLA/TPU matrix.

Figure 5b shows the Raman spectra of six SG/GR/PLA/TPU composites with different contents. There were peaks in different degrees around 1346 cm^−1^ (D-band), 1580 cm^−1^ (G-band), 2700 cm^−1^ (G′-band) and 2930 cm^−1^. Among them, the D-band indicated the defect and disorder of lattice, G-band indicated the tensile vibration of carbon atoms sp^2^ hybridization, and G′-band indicated the number of layers of graphite sheets (The peak shape moves to the right when there are multiple layers). Generally speaking, the smaller the I_D_/I_G_ value (the strength ratio of D-band to G-band), the greater the graphitization degree [16]. Therefore, the defect of 5% GR/PLA/TPU composites was the most serious, and the graphitization degree of (4% SG + 1% GR)/PLA/TPU composites was the highest. The stretching vibration peak of C-H at 930 cm^−1^ increased with the increase of I_D_/I_G_ value.

### 3.2. Effect of SG/GR Ratio on Microwave Absorbing Properties of PLA/TPU Composites

#### 3.2.1. Dielectric Loss of Composites

**Figure 6:**
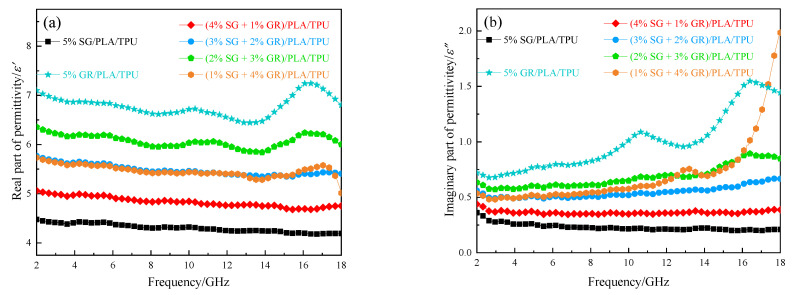

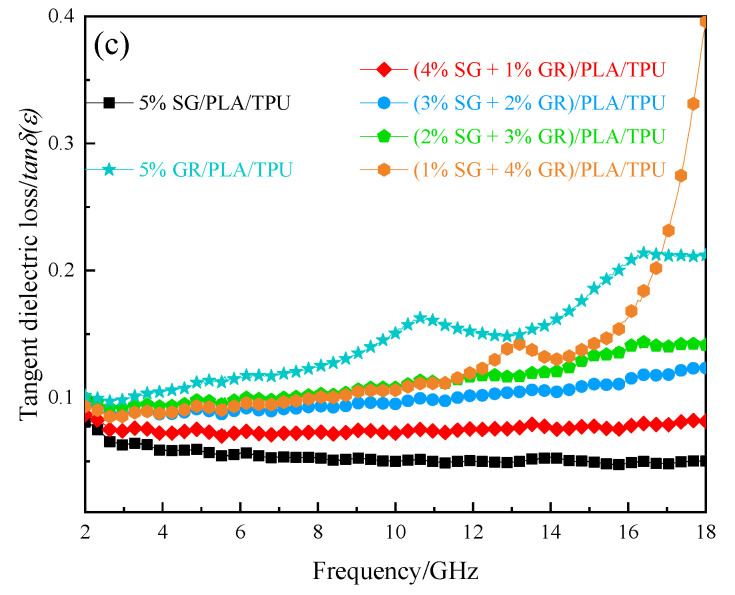
Electromagnetic parameters of six kinds of SG/GR/PLA/TPU composites with different contents: (**a**) real part of permittivity; (**b**) imaginary part of permittivity; (**c**) tangent dielectric loss.

It is nonmagnetic with SG and GR, and the electromagnetic parameters of the composite material were mainly complex dielectric constants. The curves of its real part *ε*′, imaginary part *ε*″ and tangent tan*δ* (tan*δ* = *ε*″/*ε*′) with frequency in 2~18 GHz are shown in Figure 6. Except for the (1% SG + 4% GR)/PLA/TPU composites, with the decrease of SG content and the increase of GR content, the values of *ε*′ and *ε*″ gradually increased and fluctuated obviously. It shows that the ability to store and lose charge is enhanced. When the ratio of SG/GR was 1:4, the composite of binary absorbent in PLA/TPU matrix reached the percolation threshold [17,18]. Overall, its complex dielectric constant was similar to that of (2% SG + 3% GR)/PLA/TPU composites. However, the imaginary part and tangent value suddenly rose in the range of 16~18 GHz, resulting in a large frequency response, which could explain this point.

**Figure 7:**
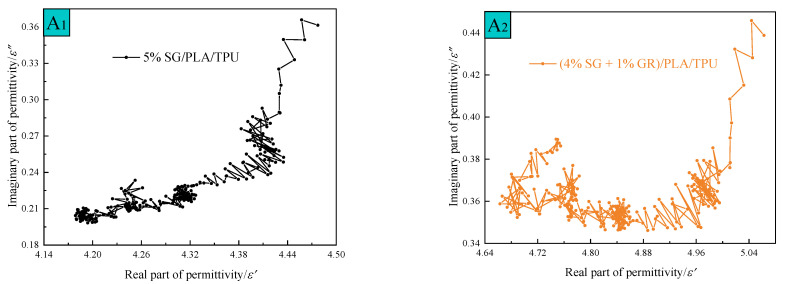

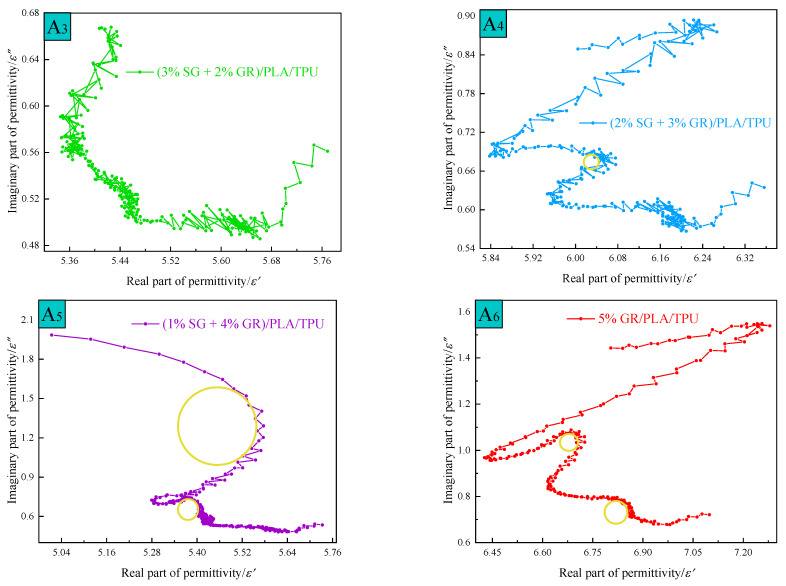
Cole–Cole curves of A_1_~A_6_ composites.

Dielectric materials are mainly characterized by interfacial polarization, dipole polarization loss and conductance loss in the microwave absorption frequency band [19,20], which are generally characterized by the Debye equation [21]:

(1) $${\left[\varepsilon^{\prime }-\frac{1}{2}\left(\varepsilon_{s}+\varepsilon_{\infty }\right)\right]}^{2}+{\left(\varepsilon^{\prime \prime }\right)}^{2}=\frac{1}{4}{\left(\varepsilon_{s}+\varepsilon_{\infty }\right)}^{2}$$

where *ε*′ is the real part of the complex dielectric constant, *ε*″ is the imaginary part of the complex dielectric constant, $\varepsilon_{s}$ is the static dielectric constant, and $\varepsilon_{\infty }$ is the high-frequency limit dielectric constant. The Cole–Cole semicircle can be obtained in the complex plane with *ε*′ as the abscissa and *ε*″ as the ordinate, as shown in Figure 7. With the decrease of SG content and the increase of GR content in A_1_~A_6_ composites, the Cole–Cole curve changed from disorder to linear order, and the formation of a semicircle was gradually observed, which meant the enhancement of dielectric loss. This shows that the GR two-dimensional sheet is more beneficial to electromagnetic wave attenuation consumption than the SG three-dimensional spherical sheet. At the same time, based on the nanosize effect [22] that GR has a smaller particle size and larger specific surface area than SG, which increases the activity of materials. The wrinkles on its surface aggravate the interfacial polarization and multiple scattering [23]. In addition, the GR retained a large number of oxygen-containing functional groups because of the redox process in the preparation, which formed defects and acted as a polarization center to induce dipole polarization [24].

#### 3.2.2. Microwave Absorption of Composites

**Figure 8:**
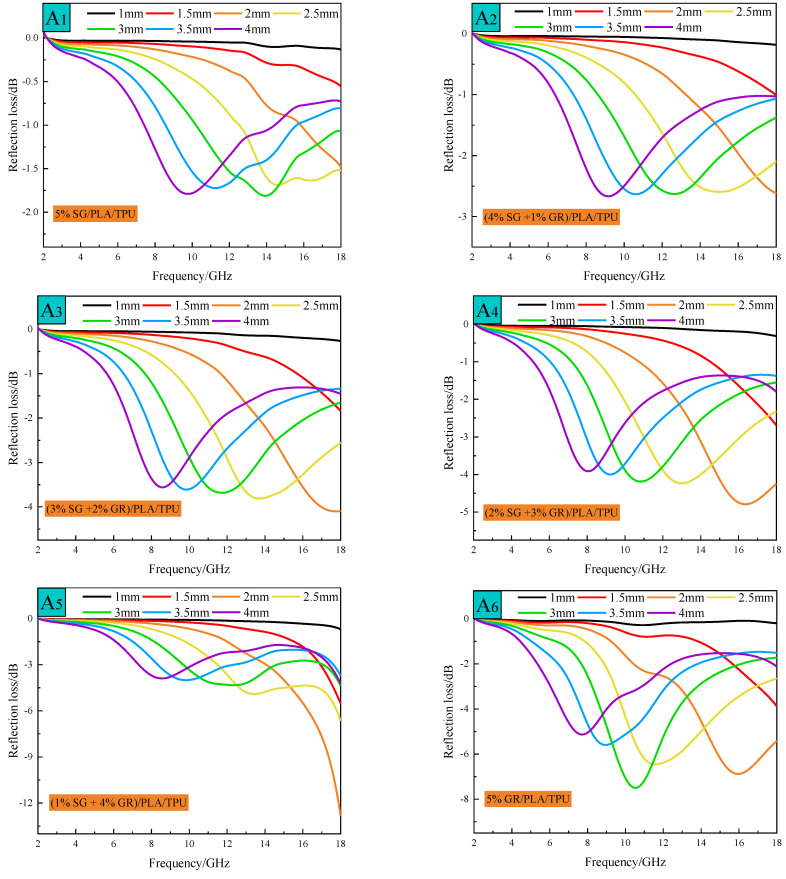
Variation curves of reflectivity with frequency of A_1_~A_6_ composites with different thicknesses.

In order to further analyze the influence of SG/GR ratio on the absorbing effect of composite materials, the reflection loss *R*_L_ of a single-layer homogeneous absorber with different thicknesses was calculated according to Formulas (2) and (3) by using transmission line theory [25,26] (the results are shown in Figure 8):

(2) $$R_{L}=20\mathrm{lg}\left|\frac{Z_{\mathrm{in}}-1}{Z_{\mathrm{in}}+1}\right|$$

(3) $$Z_{\mathrm{in}}=Z_{O}\sqrt{\frac{\mu_{r}}{\varepsilon_{r}}}\tan h\left(j\frac{2\pi fd}{c}\sqrt{\mu_{r}\varepsilon_{r}}\right)$$

where *Z*_O_ is the wave impedance of free space, *Z*_in_ is the input impedance, c is the propagation speed of electromagnetic waves in free space, *f* is the electromagnetic wave frequency, *d* is the thickness of the sample, j is the imaginary unit, *ε_r_* and *μ_r_* represent complex permittivity and complex permeability, respectively.

The reflectivity peaks of A_1_~A_6_ composites all moved to low frequency with the increase of absorbing thickness, which can be explained by quarter-wave theory and Formula (4) [27,28,29].

(4) $$f_{m}=\frac{\left(2n+1\right)c}{4d\sqrt{\varepsilon \mu }}$$

where *n* is a positive integer, c is the speed of light, *f_m_* is the peak frequency, *d* is the absorbing thickness, *ε* and *μ* represent complex permittivity and complex permeability, respectively.

**Figure 9:**
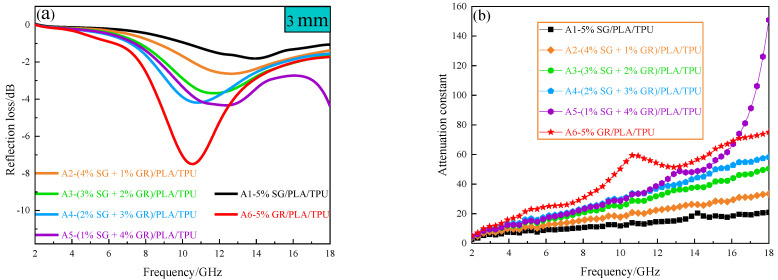
A_1_~A_6_ composites: (**a**) the curve of reflectivity with frequency at 3 mm thickness; (**b**) curve of attenuation constant with frequency.

A_6_ composite has the maximum absorption peak, reaching −7.66 dB (at 10.64 GHz). A_5_ composite has the maximum reflection loss of −12.84 dB (at 18 GHz), but there is no maximum peak in 2~18 GHz. Figure 9a is the curve of reflectivity of SG/GR/PLA/TPU composites with different contents with frequency at the thickness of 3 mm. With the decrease of SG/GR ratio (from A_1_ to A_6_), the absorption peak gradually increases and tends to move to low frequency (except A_5_ composite). It can be seen from Formula (4) that when *d* is constant, the larger $\sqrt{\varepsilon \mu }$ is, the smaller *f_m_* is. According to the electromagnetic parameters of the above composites, the correctness of this phenomenon can be proved. Figure 9b is the curve of attenuation constant of SG/GR/PLA/TPU composites with different contents with frequency, which can reflect its attenuation loss ability to electromagnetic waves. It can be calculated by Formula (5) [30]:

(5) $$\alpha =\frac{\sqrt{2}\pi f}{c}\sqrt{\mu^{\prime \prime }\varepsilon^{\prime \prime }-\mu^{\prime }\varepsilon^{\prime }+\sqrt{{\left(\mu^{\prime \prime }\varepsilon^{\prime \prime }-\mu^{\prime }\varepsilon^{\prime }\right)}^{2}+{\left(\mu^{\prime }\varepsilon^{\prime \prime }+\mu^{\prime \prime }\varepsilon^{\prime }\right)}^{2}}}$$

with the increase of frequency, the attenuation ability of A_1_~A_6_ composites increases. With the decrease of SG/GR ratio (from A_1_ to A_6_), the attenuation ability is improved and, especially, the (1% SG + 4% GR)/PLA/TPU composite increased significantly in 16~18 GHz.

**Figure 10:**
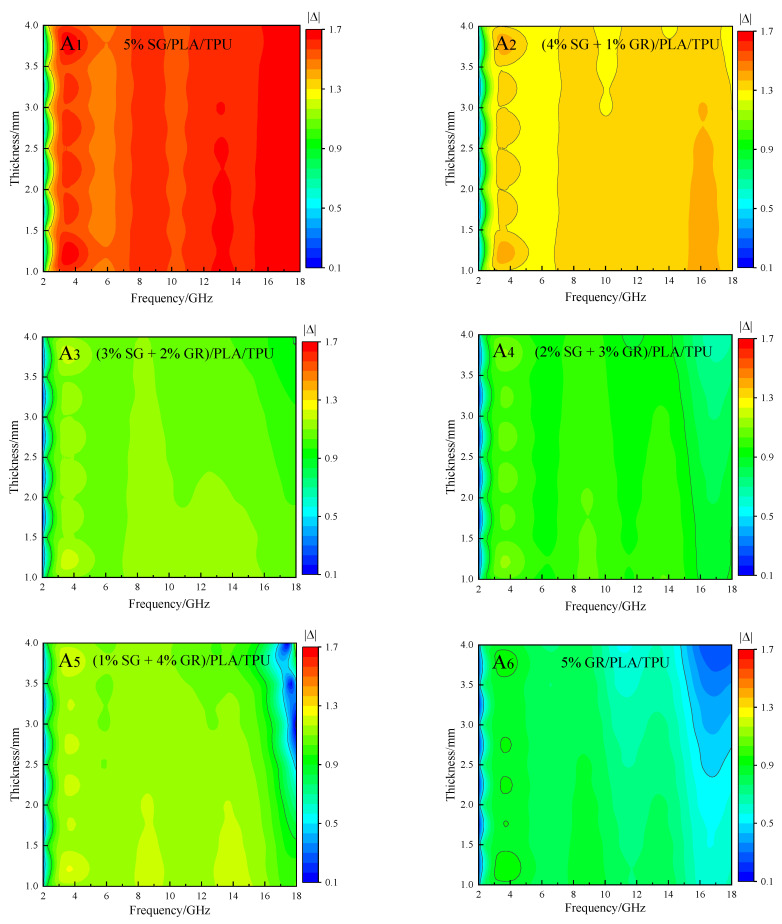
|Δ| value of A_1_~A_6_ composites.

A good wave-absorbing effect requires not only excellent attenuation ability, but also impedance matching, so as to prevent electromagnetic waves from being directly reflected into the air without incident into the material for consumption [31]. The delta function method [32] is generally used to characterize impedance matching (the smaller the value, the better the impedance matching [33]). Its calculation formulas are as follows (6), (7) and (8), and the results are shown in Figure 10.

(6) $$K=\frac{4\pi \sqrt{\mu^{\prime }\varepsilon^{\prime }}\sin\left[\left(\delta_{e}+\delta_{m}\right)/2\right]}{c\cdot \cos\delta_{e}\cdot \cos\delta_{m}}$$

(7) $$M=\frac{4\mu^{\prime }\varepsilon^{\prime }\cos\delta_{e}\cdot \cos\delta_{m}}{{\left(\mu^{\prime }\cos\delta_{e}-\varepsilon^{\prime }\cos\delta_{m}\right)}^{2}+{\left[\tan\left(\delta_{m}/2-\delta_{e}/2\right)\right]}^{2}{\left(\mu^{\prime }\cos\delta_{e}+\varepsilon^{\prime }\cos\delta_{m}\right)}^{2}}$$

(8) $$\left|∆\right|=\left|\sin h^{2}\left(Kfd\right)-M\right|$$

Overall, the 5% SG/PLA/TPU composite had the worst impedance matching (more red areas), and the 5% GR/PLA/TPU composite had the best impedance matching (more green and blue areas). For the SG/GR double-carbon absorbent, the addition of GR can improve the impedance matching. When the ratio of SG/GR was 2:3, the impedance matching in the whole frequency band with the thickness of 1~4 mm could be maintained well (more green areas). However, when the ratio of SG/GR was 1:4, their recombination reached the percolation threshold, so that good impedance matching only concentrated in the high frequency (16~18 GHz) with the thickness of 2~4 mm (blue area).

### 3.3. Effect of SG/GR Ratio on Mechanical Properties of PLA/TPU Composites

**Figure 11:**
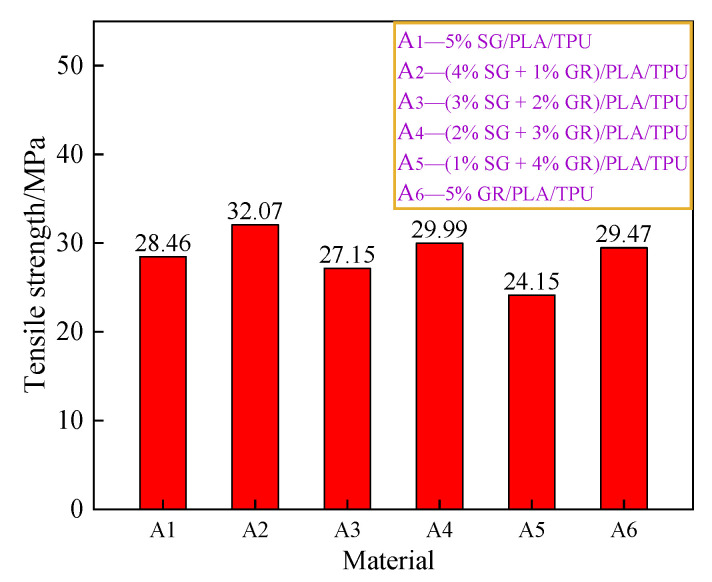
Tensile strength of A_1_~A_6_ composites.

**Figure 12:**
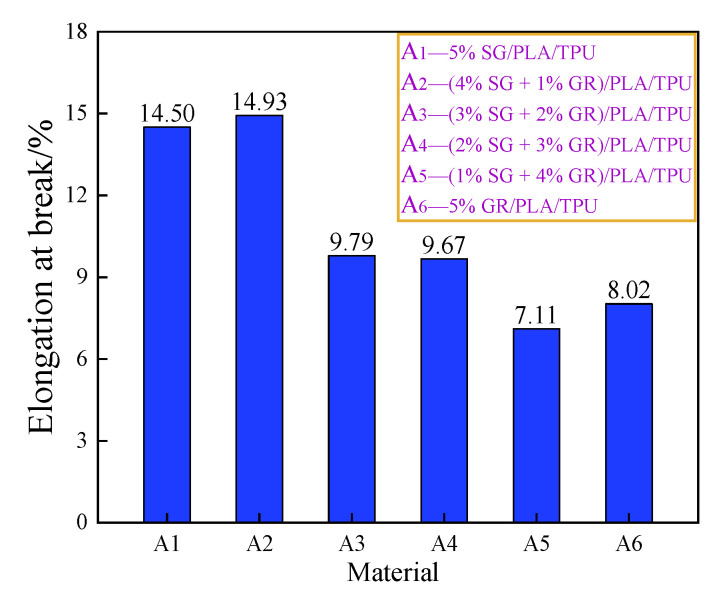
Elongation at break of A_1_~A_6_ composites.

Figure 11 and Figure 12 are the average values of tensile strength and elongation at break of the SG/GR/PLA/TPU composites with different contents. For single filler particles, such as SG or GR (A_1_, A_6_), when the content was 5%, the tensile strength of GR/PLA/TPU composite was slightly higher than that of the SG/PLA/TPU composite. However, its low elongation at break led to poor toughness. For binary filler particles (A_2_, A_3_, A_4_, A_5_), the graphitization degree of the composite was high when the ratio of SG/GR was 4:1, and tensile strength and elongation at break reached 32.07 MPa and 14.93%, respectively. At this time, it not only surpassed the single composite A_6_ in strength, but also exceeded the single composite A_1_ in toughness. The composite reached the percolation threshold when the ratio was 1:4, with a tensile strength and elongation at break of only 24.15 MPa and 7.11%, respectively.

**Figure 13:**
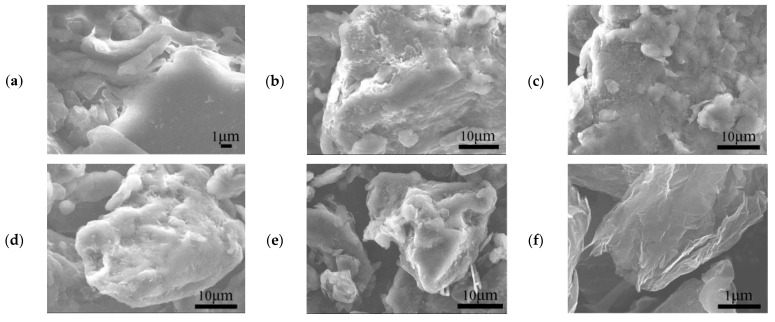
SEM diagram of A_1_~A_6_ composite powders: (**a**) 5% SG/PLA/TPU; (**b**) (4% SG + 1% GR)/PLA/TPU; (**c**) (3% SG + 2% GR)/PLA/TPU; (**d**) (2% SG + 3% GR)/PLA/TPU; (**e**) (1% SG + 4% GR)/PLA/TPU; (**f**) 5% GR/PLA/TPU.

Generally speaking, the filler particles whose shape, particle size and amount change their interfacial bonding and dispersion in the matrix, will seriously affect the mechanical properties of composites [34,35]. Figure 13 is the SEM picture of SG/GR/PLA/TPU composite powders with different content ratios. Comparing (a) with (f), three-dimensional SG particles are mainly embedded between PLA and TPU to form an “island structure”, which makes it well dispersed because of this random distribution. However, two-dimensional GR nanosheets are mainly wrapped and adhered to PLA and TPU to form a “core-shell structure”, which is beneficial to improving the interfacial adhesion, but easy to cause agglomeration. Therefore, this is the direct reason that its composites have high tensile strength but low elongation at break. Compared with (b), (c), (d) and (e), the “island” phase is transformed into a “core-shell” phase with the decrease of SG content and the increase of GR content, which makes the dispersion effect reduce greatly to cause a decline in toughness. However, when a small amount (1%) of GR is added, it not only retains the good dispersibility of SG, but also forms a hydrogen bond with the TPU hard segment through the hydrophobic effect of PLA. This improves the interfacial bonding strength by inducing the molecular chains to diffuse each other [14,36].

## 4. Conclusions

Table 5 shows the comparison of the properties between carbon-based absorbent particles and resin matrix composites in recent years. Combined with the research work in this paper, the conclusions and prospects are as follows:

1. When the content of GR in SG/GR ratio is high, GR forms a number of “core-shell structures” in the PLA/TPU matrix. On the one hand, GR whose surface wrinkles, functional groups and defects enhance the electromagnetic wave attenuation ability (polarization loss and conductivity loss) by promoting polarization (interface polarization and dipole polarization) and multiple scattering. On the other hand, it is beneficial to improve impedance matching to improve the wave absorbing effect. Among them, when the ratio of SG/GR is 1:4, the recombination reaches the percolation threshold. At this time, there is the maximum reflection loss that is −12.84 dB (at 18 GHz), which is slightly higher than that of reference [37] but lower than that of reference [38].
2. When the content of SG in SG/GR ratio is high, SG forms a large number of “island structures” in the PLA/TPU matrix, showing good dispersibility to improve the mechanical properties. Among them, when the ratio of SG/GR is 4:1, the graphitization degree of the composites is high. At this time, the maximum tensile strength and elongation at break are 32.07 MPa and 14.93%, respectively, which are higher than those in reference [39] but lower than the toughness of reference [40].
3. When the ratio of SG/GR is moderate (2:3 or 3:2), SG/GR/PLA/TPU composites have a certain electromagnetic wave-absorption capacity on the basis of high strength and toughness.
4. Compared with the single performance (wave-absorbing property or mechanical property) of carbon-based composites in Table 5, the combination of a multi-component absorbent (multiloss mechanism: electrical loss and magnetic loss) and multi-component matrix (high-strength and high-toughness matrix or particle-reinforced matrix) is the development trend for new carbon-based wave-absorbing materials to achieve the synergy of wave absorbency and mechanical properties. In addition, FDM 3D printing has obvious advantages in energy saving, environmental protection and complex structure manufacturing.

## Figures and Tables

**Table 1:** Raw material.

Name	Trademark	Preparation Method/Average Particle Size	Source
PLA	4032D	The average particle size is 62 μm	NatureWorks Inc. (Minnetonka, MN, USA).
TPU	5377A	The average particle size is 114 μm	Bayer company (Leverkusen, Germany)
SG	-	Coarse crushing, trimming, magnetic separation and high-temperature purification of natural flake graphite.	Qingdao Xinghe graphite company (Qingdao, China)
GR	-	Graphite oxidation reduction process	Yichang Xincheng graphite company (Yichang, China)

**Table 2:** Experimental instruments.

Name	Model	Manufacturer
Electrothermal constant-temperature blast drying oven	BPG-43BG	Guangzhou Bilang instrument Co., Ltd. (Guangzhou, China)
Horizontal planetary ball mill	QM-WX4	Nanjing Nanda Instrument Co., Ltd. (Nanjing, China)
Single screw extruder	SHSJ-25	Dongguan Songhu machinery Co., Ltd. (Dongguan, China)
Double nozzle printer	Allcct Tank	Wuhan Allcct Co., Ltd. (Wuhan, China)

**Table 3:** Composition of SG/GR/PLA/TPU composite wires.

Number	Name	Content (wt%)					
		Ratio	SG	GR	PLA	TPU	Ratio
A_1_	5% SG/PLA/TPU	5:0	5	0	85.5	9.5	9:1
A_2_	(4% SG + 1% GR)/PLA/TPU	4:1	4	1	85.5	9.5	9:1
A_3_	(3% SG + 2% GR)/PLA/TPU	3:2	3	2	85.5	9.5	9:1
A_4_	(2% SG + 3% GR)/PLA/TPU	2:3	2	3	85.5	9.5	9:1
A_5_	(1% SG + 4% GR)/PLA/TPU	1:4	1	4	85.5	9.5	9:1
A_6_	5% GR/PLA/TPU	0:5	0	5	85.5	9.5	9:1

**Table 4:** Testing and characterization methods of material properties.

Name	Model	Manufacturer	Test Parameters and Scope	Characterization
Specific surface and aperture analyzer	Novatouch	Quantachrome (Boynton Beach, FL, USA)	Accuracy of pressure sensor < 0.1%. The A/D converter signal resolution reaches 24 bit.	Specific surface area and pore diameter of GR
Laser particle size analyzer	S3500	Microtrac, America (Montgomeryville, PA, USA)	Measuring range: 0.02–2000 μm. Analysis accuracy: error ≤ 0.6%. Repeatability: error ≤ 1%.	Particle size distribution of SG
Field scanning electron microscope	JSM-7500F	Japan electronics Co., Ltd. (Tokyo, Japan)	Resolution: 1.0 nm (15 KV), 1.4 nm (1 KV). Magnification: 250–1,000,000 times.	Microstructure of SG, GR and their composite powders
Field high resolution transmission electron microscope	JEOL-F200	Japan electronics Co., Ltd. (Tokyo, Japan)	Resolution: 0.10 nm (TEM mode), 0.14 nm (STEM mode). Magnification: 20–2,000,000 times.	Lattice structure of GR
Fourier transform infrared spectrometer	Spectrum 100	Perkin Elmer, America (Waltham, MA, USA)	The wave number is in the range of 4000~450 cm^−1^.	Chemical structure and functional groups of SG and GR
X-ray diffractomer	Ultima IVXRD	Japan Neo-Confucianism Corporation (Tokyo, Japan)	With copper target Ka ray, scanning speed 5 (°)/min, step distance 0.02°, diffraction scanning angle 2θ = 5~80°.	Element composition and crystal type of composites
Laser confocal Raman spectrometer	Thermo Scientific DXR	Thermo Fisher Scientific (Waltham, MA, USA)	Raman shift is in the range of 100~3500 cm^−1^.	Graphitization degree of composites
Vector network analyzer	R&S ZNA	Rhodes & Schwartz Co., Ltd. (Munich, Germany)	Test the electromagnetic parameters of coaxial ring at 2~18 GHz.	Electromagnetic properties of composites
Electro mechanical universal testing machines	5569	Instron, America (Norwood, MA, USA)	The tensile rate is 2 mm/min, and each group of tensile samples is measured for 3 times and averaged.	Tensile strength and elongation at break of composites

**Table 5:** Comparison of properties between carbon-based absorbent particles and resin matrix composites in recent years.

Material	Absorbent	Matrix	Content	Absorption Maximum/dB	Tensile Strength/MPa	Elongation at Break/%	Ref.
CB/EP	Carbon black	Epoxy resin	5%	−10	-	-	[37]
GR/FeNi50/PLA	Graphene/FeNi50 (magnetic)	Polylactide	5%	−20.8	-	-	[38]
GR/PLA	Graphene	Polylactide	5%	-	21.4	3.8	[39]
PP/POE/CB	Carbon black	Polypropylene/polyoxyethylene	5%	-	16.8	650	[40]

## Data Availability

Not applicable.
